# Effect of Juice and Extracts from *Saposhnikovia divaricata* Root on the Colon Cancer Cells Caco-2

**DOI:** 10.3390/ijms20184526

**Published:** 2019-09-12

**Authors:** Magdalena Matusiewicz, Katarzyna Barbara Bączek, Iwona Kosieradzka, Tomasz Niemiec, Marta Grodzik, Jarosław Szczepaniak, Sylwia Orlińska, Zenon Węglarz

**Affiliations:** 1 Department of Animal Nutrition and Biotechnology, Faculty of Animal Sciences, Warsaw University of Life Sciences, Ciszewskiego 8, 02-786 Warsaw, Poland; iwona_kosieradzka@sggw.pl (I.K.); tomasz_niemiec@sggw.pl (T.N.); marta_grodzik@sggw.pl (M.G.); jaroslaw_szczepaniak@sggw.pl (J.S.); sylwia.orzelek@gmail.com (S.O.); 2 Laboratory of New Herbal Products, Department of Vegetable and Medicinal Plants, Faculty of Horticulture, Biotechnology and Landscape Architecture, Warsaw University of Life Sciences, Nowoursynowska 159, 02-776 Warsaw, Poland; katarzyna_baczek@sggw.pl (K.B.B.); zenon_weglarz@sggw.pl (Z.W.)

**Keywords:** *Saposhnikovia divaricata*, juice, extract, chemical composition, colon cancer, viability, apoptosis

## Abstract

Colorectal cancer ranks 3rd in terms of cancer incidence. Growth and development of colon cancer cells may be affected by juice and extracts from *Saposhnikovia divaricata* root. The objective of the research was to analyze the effect of *S. divaricata* juice and extracts on the viability, membrane integrity and types of cell death of Caco-2 cells. Juice and extracts were analyzed using Ultra-High Performance Liquid Chromatography-Mass Spectrometry (UHPLC-MS) and in respect of the presence of antioxidants, total carbohydrates, protein, fat and polyphenols. The contents of cimifugin β-D-glucopyranoside, cimifugin, 4′-O-glucopyranosyl-5-O-methylvisamminol, imperatorin and protein were the highest in juice. 50% Hydroethanolic extract had the greatest antioxidant potential, concentration of polyphenols and fat. Water extract was characterized by the highest content of glutathione. Juice and 75% hydroethanolic extract contained the most carbohydrates. After the application of juice, 50% extract and the juice fraction containing the molecules with molecular weights >50 kDa, a decrease of the cell viability was noted. Juice and this extract exhibited the protective properties in relation to the cell membranes and they induced apoptosis. The knowledge of further mechanisms of anticancer activity of the examined products will allow to consider their use as part of combination therapy.

## 1. Introduction

Globally colorectal cancer ranks 3rd in terms of cancer incidence and 2nd in terms of mortality [1]. The incidence rates of this disease in transition countries are over 3-fold higher than in transitioning countries and the mortality rates are over 2-fold higher. The highest incidence rates of colon cancer were noted in European countries, Australia and New Zealand, North America, eastern Asia and Uruguay. 

The predisposition to the development of colorectal cancer is hereditary, with a risk of about 12–35% attributed to the genetic factors [2]. The increase in colorectal cancer incidence is related to the effects of dietary patterns, obesity and lifestyle factors and the risk of this cancer can be reduced through proper dietary habits and lifestyle [2,3]. 

Current treatment of colorectal cancer is based on surgery with or without chemotherapy and/or radiotherapy, before or after surgery [4]. However, this treatment is not always effective because of the development of recurrent disease in 30% of patients with stage I-III colorectal cancer and up to 65% of patients with stage IV [5]. The adverse effects of this therapy impair the patient′s life quality and may negatively affect the treatment course, outcome and costs [2].

A promising approach in minimizing the adverse effects of conventional therapy could be the combination therapy using both a conventional chemotherapeutics or radiotherapy and natural compound(s) with significant pharmacological effects derived from plant, marine or microorganism sources [2,4,6]. The combination therapy targets multiple metabolic pathways using different mechanisms to decrease the development of resistance to anticancer drugs, increasing the sensitivity to the action of the chemotherapeutics [7]. Recent studies have highlighted the importance of combination therapy and consider it more effective compared to conventional chemotherapeutic agents [8]. Some natural compounds are able to modulate signaling pathways and the expression of genes associated with cell proliferation, differentiation, apoptosis, angiogenesis and metastasis [9].

In Chinese and Japanese traditional medicine, the underground organs of *Saposhnikovia divaricata* (Turcz.) Schischk. (*Apiaceae*) have been used as one of the most important drugs [10]. The species grows wild in north-eastern China and Inner Mongolia [11,12]. *Saposhnikoviae Radix* is officially listed in the pharmacopoeias of China, Japan and Korea and is also one of the candidates for inclusion in the German Pharmacopoeia (DAB) [13,14]. 

Extracts from this raw material have been used for the treatment of common cold, adiaphoresis, cough, allergic rhinitis, headache, migraine, dizziness as well as in rheumatic disorders, arthralgia, diarrhea, smallpox and anxiety in children [10,15,16,17]. They have demonstrated anti-inflammatory, anti-pyretic, anti-convulsant activities and inhibitory effects on peptic ulcers. Antimicrobial, antiviral, antioxidant and antiproliferative activities were also mentioned [10,18]. 

Two compounds, cimifugin β-D-glucopyranoside and 4′-O-glucopyranosyl-5-O-methyl-visamminol, are considered to be mainly responsible for the activity of this raw material. These chromones were selected as reference compounds in the identification and quality assessment of *Saposhnikoviae Radix*. According to the Chinese Pharmacopoeia (2010) [19], the raw material (dry matter) should contain not less than 0.24% of both compounds. Aside from chromones, *Saposhnikoviae Radix* is rich in coumarins, furanocoumarins, polyacetylenes and polysaccharides. It also contains some phenolics, acid esters and a small amount of essential oil, with caryophyllene oxide, sabinene and β-pinene as dominant compounds [15,20,21,22].

Cimifugin, a coumarin abundant in *S. divaricata* roots, exerts analgesic effects [20]. This effect was also confirmed for 4′-O-glucopyranosyl-5-O-methylvisamminol and cimifugin β-D-gluco-pyranoside, as well as antipyretic, anti-platelet aggregation and anti-inflammatory actions [23]. Cimifugin β-D-glucopyranoside and cimifugin appear to inhibit major inflammatory pathways, nuclear factor (NF)-κB, cAMP response element-binding protein (CREB), mitogen activated protein kinases (MAPKs) and nitric oxide production [15,24]. The rate of inhibition of the production of NO in lipopolysaccharide (LPS)-activated RAW264.7 macrophages by cimifugin β-D-glucopyranoside was lower than by cimifugin, its deglucosylated biotransformation product, formed as a result of incubation with human intestinal flora [25]. 4′-O-Glucopyranosyl-5-O-methylvisamminol, cimifugin β-D-glucopyranoside and cimifugin are the inhibitors of matrix metalloproteases (MMP)-2, involved in the occurrence and development of inflammatory reactions, tissue remodeling, wound healing and cancer. 4′-O-Glucopyranosyl-5-O-methylvisamminol turned out to be the strongest inhibitor [26].

The furanocoumarin imperatorin, an important constituent of *S. divaricata* root, exhibits many pharmacological properties, including anticancer, anti-inflammatory, anti-osteoporotic, myorelaxant, hepatoprotective, antibacterial and antiviral activities, in addition to a beneficial influence on the cardiovascular and central nervous system [27]. Imperatorin is contained in many traditional drugs, especially in Traditional Chinese Medicine. 

Considering the above multidirectional health-promoting properties of the root of *S. divaricata*, including anticancer activities, the juice and extracts derived from it, containing chemical compounds possessing potential anticancer properties, may affect growth and development of colon cancer cells. The objective of this research was to analyze the chemical composition of juice and water, 50% and 75% hydroethanolic extracts from the root of *S. divaricata*, as well as to determine their redox properties. Then, the effect of juice and extracts on the viability of Caco-2 human colorectal adenocarcinoma cells was evaluated. Moreover, the influence of fractions of juice and 50% hydroethanolic extract, containing components of different molecular weights, on Caco-2 cell viability was examined. Finally, the effect of juice and 50% hydroethanolic extract on the membrane integrity and types of cell death was determined.

## 2. Results

### 2.1. Content of Cimifugin β-D-glucopyranoside, Cimifugin, 4′-O-Glucopyranosyl-5-O-methylvisamminol and Imperatorin

**Figure 1:**
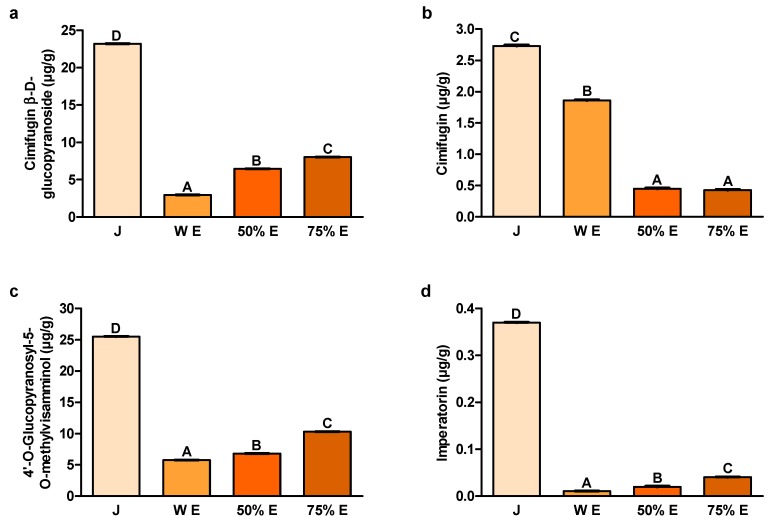
Content of (**a**) cimifugin β-D-glucopyranoside; (**b**) cimifugin; (**c**) 4′-O-glucopyranosyl-5-O-methylvisamminol and (**d**) imperatorin in juice (J), water extract (W E), 50% hydroethanolic extract (50% E) and 75% hydroethanolic extract (75% E) from *Saposhnikovia divaricata* root. Error bars indicate standard error of the mean. Statistically significant effect: values of one compound without common superscript (A,B,C,D) are significantly different (*p* < 0.01). *n* = 3.

The contents of the examined bioactive compounds were by far the highest in juice (Figure 1). The contents of cimifugin β-D-glucopyranoside (Figure 1a), 4′-O-glucopyranosyl-5-O-methyl-visamminol (Figure 1c) and imperatorin (Figure 1d) in 75% hydroethanolic extract were statistically significantly lower than in juice, but significantly higher than in 50% extract. Concentrations of these bioactives were significantly the smallest in water extract. Water extract contained significantly less cimifugin than juice but significantly more compared to both hydroethanolic extracts (Figure 1b).

### 2.2. Redox State Indicators

**Figure 2:**
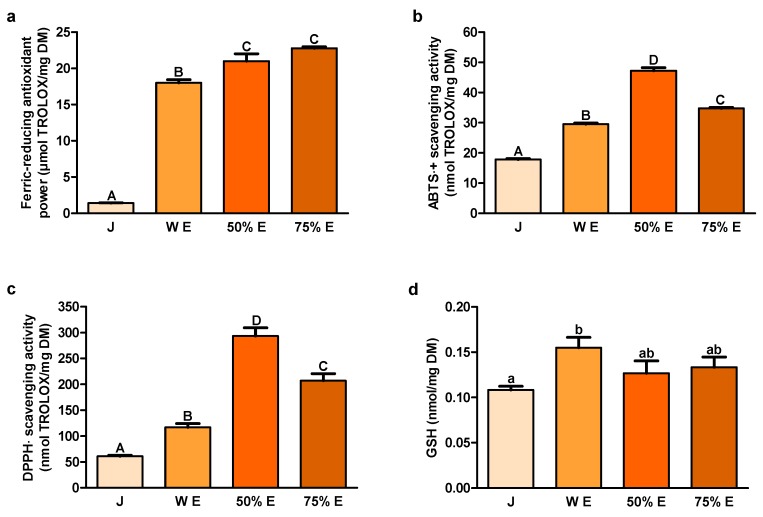
Indicators of redox state: (**a**) ferric-reducing antioxidant power; (**b**) ABTS﮲^+^ (2.2′-azino-bis(3-ethylbenzthiazoline-6-sulfonic acid) radical cation) scavenging activity; (**c**) DPPH﮲ (2.2-diphenyl-1-picrylhydrazyl radical) scavenging activity and (**d**) GSH (glutathione) in juice (J), water extract (W E), 50% hydroethanolic extract (50% E) and 75% hydroethanolic extract (75% E) from *Saposhnikovia divaricata* root. Error bars indicate standard error of the mean. Statistically significant effect: values of one indicator without common superscript are significantly different (a,b—at a significance level of *p* < 0.05; A,B,C,D—at a significance level of *p* < 0.01). *n* = 6 (Figure 2a,c,d), *n* = 5 (Figure 2b). DM—dry matter.

In the current study, the ferric-reducing antioxidant power of 50% and 75% hydroethanolic extracts was significantly greater than in water extract and its value was the lowest in juice (Figure 2a). Scavenging activities of 2.2′-azino-bis(3-ethylbenzthiazoline-6-sulfonic acid) radical cation (ABTS﮲^+^) and 2.2-diphenyl-1-picrylhydrazyl radical (DPPH﮲) by 50% hydroethanolic extract were significantly higher than by 75% extract (Figure 2b,c). Scavenging activities of ABTS﮲^+^ and DPPH﮲ by 75% extract were significantly greater than by water extract. Significantly the lowest values of this redox state indicators were noted for juice. Significantly the highest content of glutathione (GSH) was found in water extract and the lowest in juice (Figure 2d). 

### 2.3. Content of Total Carbohydrates, Protein and Fat

The data showed that juice and extracts consisted mostly of total carbohydrates, the content of which was statistically significantly the highest in 75% hydroethanolic extract and juice and significantly the lowest in 50% extract (Table 1). Furthermore, the extracts scarcely contained total protein, whereas juice contained about 5%. The total fat content was significantly higher in 50% hydroethanolic extract than in juice. 

### 2.4. Concentration of Polyphenols

The content of polyphenols was statistically significantly the highest in 50% hydroethanolic extract (Table 1). 75% Extract contained significantly less polyphenols than 50% extract and significantly more than water extract. Polyphenol concentration was significantly the lowest in juice.

### 2.5. Effect of Juice and Extracts on Cell Viability

**Figure 3:**
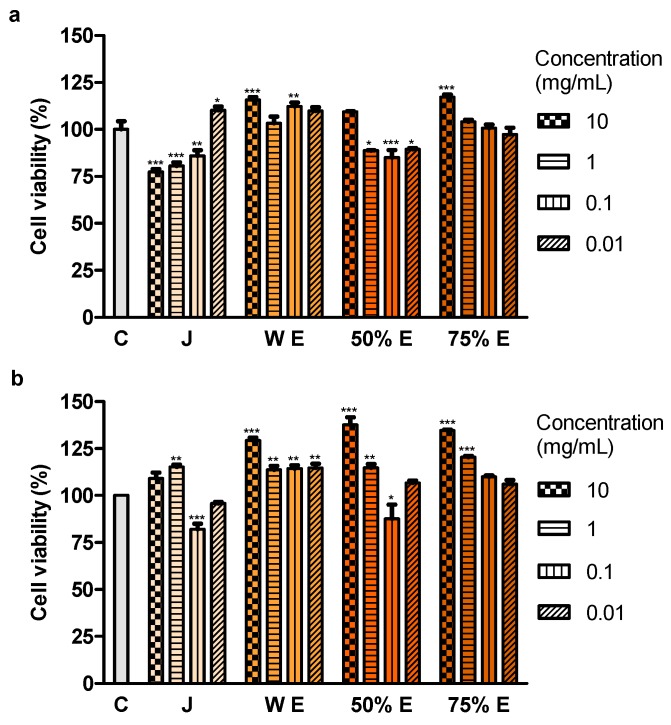
Viability of Caco-2 cells after (**a**) 24 h and (**b**) 72 h of treatment with juice (J), water extract (W E), 50% hydroethanolic extract (50% E) and 75% hydroethanolic extract (75% E) from *Saposhnikovia divaricata* root, at different concentrations. C—control cells (treated with deionized water). Error bars indicate standard error of the mean. Statistically significant effect: * represents values that differ from control at *p* < 0.05, ** represents values that differ from control at *p* < 0.01, *** represents values that differ from control at *p* < 0.001. *n* = 5.

The effect of different concentrations of juice and extracts on the viability of Caco-2 cells was assessed by a methylthiazolyldiphenyl-tetrazolium bromide (MTT) test, based on the oxidative activity of the mitochondria of live cells. The results of the 24-h assay demonstrated that three concentrations of juice (10, 1 and 0.1 mg/mL) and 50% hydroethanolic extract (1, 0.1 and 0.01 mg/mL) statistically significantly reduced cell viability (Figure 3a). The concentration of 0.1 mg/mL of juice and 50% extract was found to be effective in decreasing cell viability also in the 72-h assay (Figure 3b). No reduction of cell viability was observed after the application of water and 75% extracts.

### 2.6. Effect of Fractions of Juice and 50% Hydroethanolic Extract on Cell Viability

**Figure 4:**
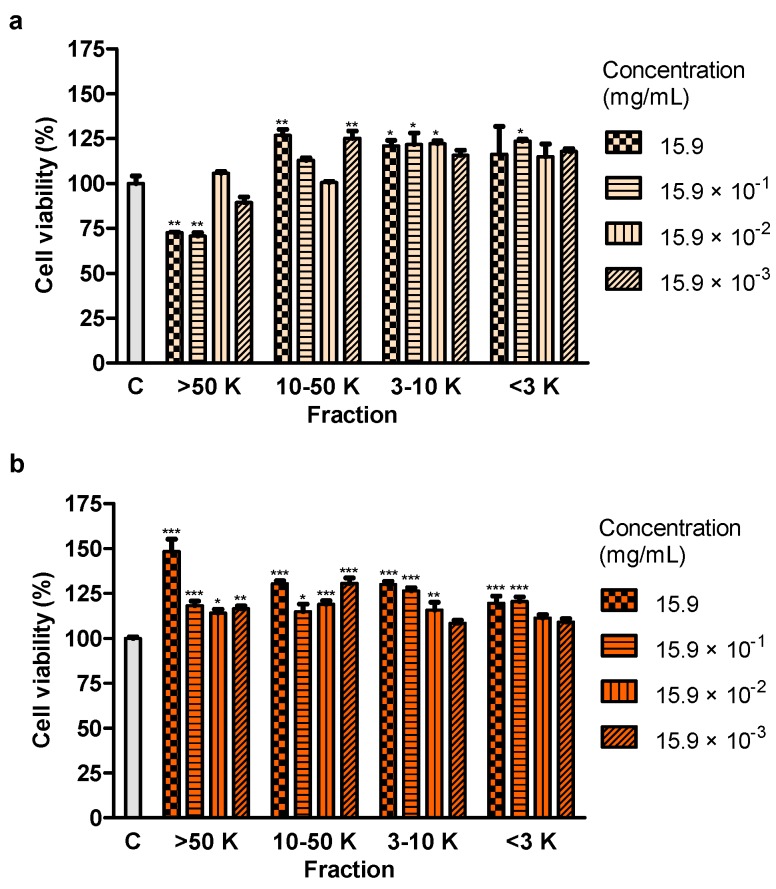
Viability of Caco-2 cells after treatment for 24 h with fractions >50 kDa (>50 K), 10–50 kDa (10–50 K), 3-10 kDa (3–10 K) and <3 kDa (<3 K) of (**a**) juice and (**b**) 50% hydroethanolic extract from *Saposhnikovia divaricata* root, at different concentrations. C–control cells (treated with deionized water). Error bars indicate standard error of the mean. Statistically significant effect: * represents values that differ from control at *p* < 0.05, ** represents values that differ from control at *p* < 0.01, *** represents values that differ from control at *p* < 0.001. *n* = 5.

24-h test revealed that the molecules of molecular weight >50 kDa of juice (at the concentrations of 15.9 and 15.9 × 10^−1^ mg/mL) decreased the viability of Caco-2 cells in a statistically significant manner (Figure 4a). The remaining fractions, containing particles of molecular weights 10–50 kDa, 3–10 kDa and <3 kDa, as well as fractions of 50% hydroethanolic extract did not show this effect (Figure 4a,b).

### 2.7. Effect of Juice and 50% Hydroethanolic Extract on Cell Membrane Integrity

**Figure 5:**
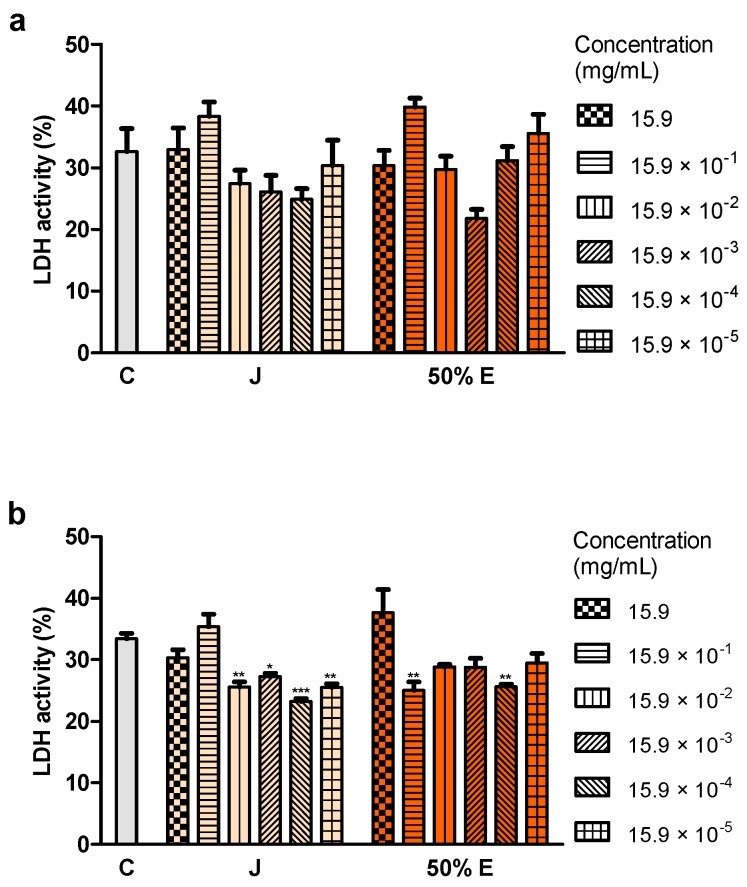
Integrity of membranes of Caco-2 cells after (**a**) 24 h and (**b**) 72 h of treatment with juice (J) and 50% hydroethanolic extract (50% E) from *Saposhnikovia divaricata* root, at different concentrations. C—control cells (treated with deionized water), LDH—lactate dehydrogenase. Error bars indicate standard error of the mean. Statistically significant effect: * represents values that differ from control at *p* < 0.05, ** represents values that differ from control at *p* < 0.01, *** represents values that differ from control at *p* < 0.001. *n* = 6.

The integrity of cell membranes after the application of juice and 50% hydroethanolic extract was examined based on the release of the cytosolic enzyme–lactate dehydrogenase (LDH) from damaged cells. LDH activity did not change statistically significantly after the treatment of the cells with juice and 50% extract for 24 h (Figure 5a). Some concentrations of juice (from 15.9 × 10^−2^ to 15.9 × 10^−5^ mg/mL) and 50% extract (15.9 × 10^−1^ and 15.9 × 10^−4^ mg/mL) significantly reduced LDH activity after the treatment for 72 h (Figure 5b). 

### 2.8. Effect of Juice and 50% Hydroethanolic Extract on Types of Cell Death

**Figure 6:**
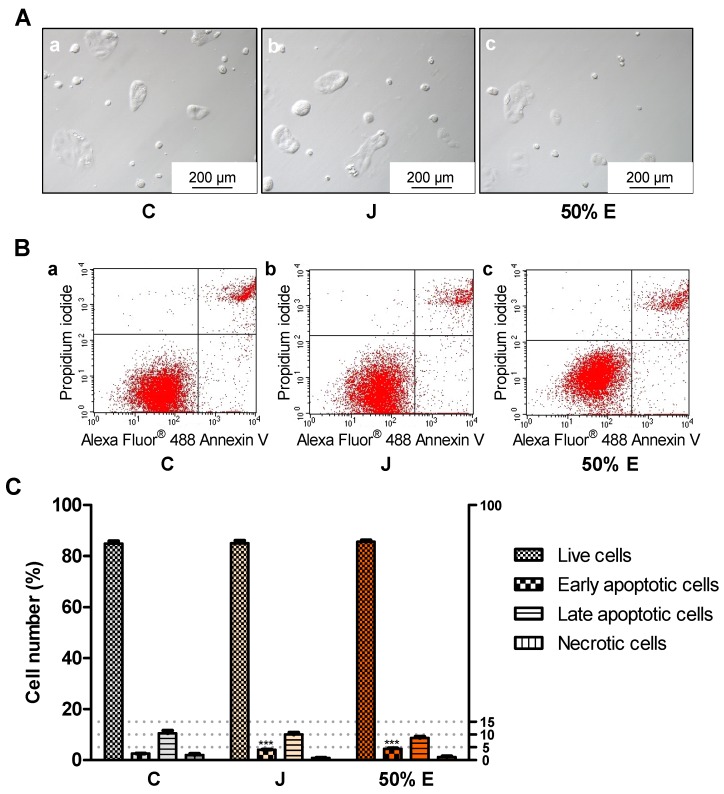
Types of death of Caco-2 cells after 24 h of treatment with juice (J) and 50% hydroethanolic extract (50% E) from *Saposhnikovia divaricata* root, at the concentration of 15.9 × 10^−1^ mg/mL. C—control cells (treated with deionized water). Panel (**A**)—images from light microscope, panel (**B**)—exemplary flow cytometry charts (lower left quadrant–live cells, lower right quadrant–early apoptotic cells, upper right quadrant–late apoptotic cells, upper left quadrant–necrotic cells), panel (**C**)—graphs with error bars indicating standard error of the mean. Statistically significant effect: *** represents values that differ from control at *p* < 0.001. *n* = 6.

**Figure 7:**
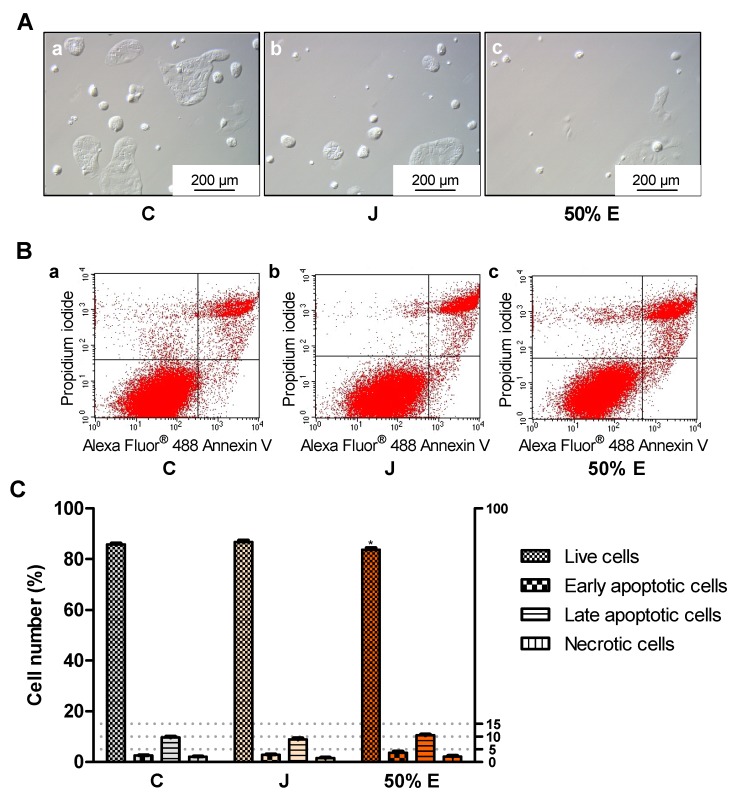
Types of death of Caco-2 cells after 72 h of treatment with juice (J) and 50% hydroethanolic extract (50% E) from *Saposhnikovia divaricata* root, at the concentration of 15.9 × 10^−2^ mg/mL. C—control cells (treated with deionized water). Panel (**A**)—images from light microscope, panel (**B**)—exemplary flow cytometry charts (lower left quadrant—live cells, lower right quadrant—early apoptotic cells, upper right quadrant–late apoptotic cells, upper left quadrant–necrotic cells), panel (**C**)—graphs with error bars indicating standard error of the mean. Statistically significant effect: * represents values that differ from control at *p* < 0.05. *n* = 6.

Incubation of Caco-2 cells with juice and 50% hydroethanolic extract (15.9 × 10^−1^ mg/mL) statistically significantly increased the number of early apoptotic cells in the 24-h test (Figure 6). Treatment with 50% extract (15.9 × 10^−2^ mg/mL) significantly reduced the number of live cells in the 72-h test (Figure 7).

## 3. Discussion

The content of cimifugin β-D-glucopyranoside, one of the main *S. divaricata* chromones, turned out to be the highest in juice. Its content in 75% hydroethanolic extract was much lower than in juice and higher than in other extracts. The anticancer activity of this compound was confirmed in the in vitro studies.

According to Zhang et al., cimifugin β-D-glucopyranoside, at concentrations equal to or higher than 10 μM (4.68 μg/mL), decreased the viability of Jurkat and J45.01 acute T cell leukemia cells, after 24 h and 48 h of treatment [28]. Jurkat cells incubated with this compound, at the concentrations of 20 μM (9.37 μg/mL) and 40 μM (18.74 μg/mL), for 24 h and 48 h, underwent apoptosis. The number of early and late apoptotic cells increased with the increase of dose applied and the extension of time of treatment. Moreover, cimifugin β-D-glucopyranoside may induce apoptosis by the activation of the caspase pathway. The results suggest that this compound blocked the cell cycle at the G2/M phase. After the incubation with 20 μM (9.37 μg/mL) cimifugin β-D-glucopyranoside, for 48 h, about 40% of cells were arrested at the G2/M phase of the cell cycle. In case of the treatment with 40 μM (18.74 μg/mL) cimifugin β-D-glucopyranoside, the number of cells at the G2/M phase was 56%, whereas only 16.7% of control cells were at this phase. This chromone may function as the inhibitor of β-tubulin and tubulin polymerization that leads to cell cycle arrest at the G2/M phase. In the study of Huyen et al., cimifugin β-D-glucopyranoside, isolated from the 70% hydroethanolic extract from the rhizome of *Cimicifuga dahurica* (Turcz.) Maxim. (*Ranunculaceae*), at the concentration of 10 μM (4.68 μg/mL), showed significant antiproliferative effect on MCF-7 breast cancer cells exposed to a proliferation stimulator [29].

The content of cimifugin, the other chromone of *S. divaricata* root, was the highest in juice. Among the extracts, the highest content of this compound was noted in the water extract. Yokosuka et al. showed that the methanolic extract from the root and rhizome of *S. divaricata* decreased the viability of HL-60 human acute promyelocytic leukemia cells, after 72 h of treatment [22]. Furthermore, six chromone derivatives (3′-O-angeloyhamaudol, ledebouriellol, divaricatol, cimifugin, sec-O-glucosylhamaudol and 5-O-methylvisammioside) and five polyacetylene derivatives were isolated from the methanolic extract. The first three chromone derivatives decreased the viability of HL-60 cells, however, there was no effect in case of the last three, including cimifugin.

The results suggest that the cells exposed to 3′-O-angeloyhamaudol underwent apoptosis. The results indicate that this compound, by the loss of the mitochondrial membrane potential, induced the cytochrome c release from the space between the mitochondrial membranes into the cytosol. In the cytosol, cytochrome c interacted with Apaf-1 (apoptotic protease-activating factor-1) and the complex of these proteins activated caspase-9, which activated caspase-3. However, the treatment of MCF-7 cells with the extract from *Cimicifuga foetida* L. (*Ranunculaceae*), containing cimifugin and cimifugin glucoside, resulted in the reduction of cell number [30]. Moreover, this extract diminished the expression of mRNA of heat shock protein 27, which content is increased in different human cancers and which exhibits cytoprotective activity, affecting tumorigenesis and tumor susceptibility to treatment.

The content of the second main *S. divaricata* chromone-4′-O-glucopyranosyl-5-O-methyl-visamminol, like cimifugin β-D-glucopyranoside, was by far the highest in juice. Among the extracts, the greatest concentration of this compound was also contained in 75% hydroethanolic extract. In the research of Ma et al., 4′-O-glucopyranosyl-5-O-methylvisamminol and four other chromone glycosides isolated from the 70% hydroethanolic extract from the root of *S. divaricata*, decreased the viability of three cancer cell lines: PC-3 human Caucasian prostate adenocarcinoma, SK-OV-3 human ovarian carcinoma and H460 human lung carcinoma [31]. 

The content of imperatorin, belonging to the furanocoumarins, was also by far the largest in juice from the root of *S. divaricata*. Its content in 75% hydroethanolic extract was also higher compared to other extracts. Numerous in vitro and in vivo studies have demonstrated the anticancer properties of imperatorin and its potential mechanisms of action. Imperatorin isolated from the extract from the medicinal plant *Angelica dahurica* (Fisch. ex Hoffm.) Benth. et Hook. f. ex Franch. et Sav. (*Apiaceae*) significantly decreased the viability of HCT-15 human colorectal adenocarcinoma, A549 human non-small cell lung cancer, SK-OV-3 human Caucasian ovary adenocarcinoma, SK-MEL-2 human malignant melanoma and XF498 human central nervous system cancer cells, after 48 h of treatment [32]. In turn, imperatorin separated from the 85% methanolic fraction from *Glehnia littoralis* F. Schmidt ex Miq. (*Apiaceae*), at the concentrations of 1, 10 and 100 μM (0.27, 2.70 and 27.03 μg/mL, respectively), exhibited dose-dependent decrease of the viability of HT-29 human colorectal adenocarcinoma cells, after 48 h of treatment [33]. Moreover, after this time of incubation, it reduced the expression of mRNA of B-cell lymphoma-2 (Bcl-2), an intracellular suppressor of apoptosis. Imperatorin decreased also the expression of mRNA of inducible nitric oxide synthase (iNOS) and cyclooxygenase-2 (COX-2), proinflammatory enzymes related to the pathophysiology of many chronic diseases and cancers. Furthermore, imperatorin from trifoliate orange, *Poncirus trifoliata* (L.) Raf. (*Rutaceae*), at the concentrations of 74 and 740 nmol/mL (20.00 and 200.01 μg/mL), decreased the viability of HCT-15 human colorectal adenocarcinoma cells and, at the concentrations of 7.4, 74 and 740 nmol/mL (2.00, 20.00 and 200.01 μg/mL, respectively), reduced the viability of SNU-449 human hepatocellular carcinoma cells, after 4 days of treatment [34]. The results suggest that after this time of treatment, with the concentrations of 74 and 740 nmol/mL (20.00 and 200.01 μg/mL), it induced apoptosis. Moreover, imperatorin, at 370 nmol/mL (100.00 μg/mL) and at 740 nmol/mL (200.01 μg/mL), induced the cell cycle arrest at the G1-SubG1 (apoptotic) phase, after 24 h of treatment. After this time, imperatorin, at the concentrations of 370 and 740 nmol/mL (100.00 and 200.01 μg/mL), upregulated the expression of Bcl-2 associated X protein (Bax), the pro-apoptotic protein and downregulated the expression of Bcl-2, the anti-apoptotic protein. In the study of Zheng et al., imperatorin, at the concentrations of 20–200 μM (5.41–54.06 μg/mL), reduced the viability of HT-29 human colorectal adenocarcinoma cells, after 24 h, 48 h and 72 h of treatment [35]. This furanocoumarin, at the concentrations of 40–200 μM (10.81–54.06 μg/mL), did not significantly influence on the level of activity of released LDH, after 48 h of treatment. After this time of treatment, imperatorin, at the concentrations of 100, 200 and 300 μM (27.03, 54.06 and 81.08 μg/mL, respectively), induced apoptosis. This compound modulated the intrinsic apoptotic pathway, involving the caspase cascade, Bcl-2 members, p53, p21 and MDM2 (mouse double minute 2 homolog), and made considerable changes in the protein expression of caspase-8, Bax, MDM2, p53 and p21. Moreover, imperatorin, at the concentrations of 50, 100 and 150 μM (13.51, 27.03 and 40.54 μg/mL, respectively), induced cell cycle arrest at the G1 phase. This furanocoumarin also increased the content of reactive oxygen species (ROS) in the cells, after 24 h of treatment. According to Mi et al., imperatorin from the root of *Angelica dahurica* inhibited the activation of hypoxia-induced HIF-1 (hypoxia-inducible factor-1) [36]. It downregulated the protein expression of HIF-1α and the expression of HIF target genes: vascular endothelial growth factor (VEGF) and erythropoietin, essential for tumor growth. Imperatorin suppressed the synthesis of HIF-1α protein by the inhibition of the mammalian target of rapamycin (mTOR)/ribosomal protein S6 kinase (p70S6K)/eIF4E binding protein-1 (4E-BP1) and mitogen-activated protein kinase (MAPK) pathways. Moreover, this compound arrested the cell cycle at the G1 phase. It also inhibited tumor growth and blocked tumor angiogenesis in a murine xenograft model, without apparent toxicity.

The bioavailability of cimifugin β-D-glucopyranoside depends to a large extent on its biotransformation in the colon [25]. Cimifugin, as most of the examined chromones and coumarins of *S. divaricata*, turned out to be well-absorbed in the Caco-2 monolayer model, whereas cimifugin β-D-glucopyranoside was moderately absorbed [37]. The passive diffusion mechanism for these compounds was proposed. According to Li et al., cimifugin β-D-glucopyranoside exhibited low bioavailability and was rapidly eliminated after oral administration [38]. Moreover, absorption and elimination of cimifugin β-D-glucopyranoside and cimifugin in the root extract of *S. divaricata* were longer than for the single compounds, probably because of the interactions between the chemical compounds in the extract. The experiment on rats of Gao and Luan demonstrated that imperatorin is mainly absorbed in the colon [39]. The absorption mechanism may be the passive diffusion with the active transport. Well absorption in the colon of the most of the chromones and coumarins of *S. divaricata* and the positive influence on the absorption of the interaction between the compounds in *S. divaricata* extract indicate that the examined juice and extracts, after ingestion, may to a large extent influence on the colon cells, better than single compounds.

Long-term ingestion of antioxidants could decrease the colorectal cancer incidence in rats, by reducing oxidative stress [40]. These compounds could also improve the severity of colitis, the inflammatory and the preneoplastic state of the colorectal cancer, by reducing the elevated content of malondialdehyde (MDA). In our study, ferric-reducing antioxidant power, based on the electron donation by antioxidants to neutralize free radicals by forming stable products [41], was the highest for hydroethanolic extracts from the root of *S. divaricata* and the lowest for juice. Scavenging activity of ABTS﮲^+^, also representing the electron transfer properties, and scavenging activity of DPPH﮲, a stable free radical accepting an electron or a hydrogen to become a stable molecule, were the highest for 50% hydroethanolic extract. The values of these indices were lower for 75% hydroethanolic extract and water extract and the lowest for juice. ABTS﮲^+^ scavenging activity by 70% hydroethanolic extract from the root of *S. divaricata* was demonstrated earlier by other authors [18]. Our results indicate the highest content of hydrophilic and hydrophobic antioxidants in 50% hydroethanolic extract and the largest potential of this extract in decreasing oxidative stress in the organism. 

Among the enzymatic systems responsible for the intracellular redox balance maintenance, the main role plays GSH, which participates in antioxidant defense systems and many metabolic processes [42,43]. On the other hand, elevated GSH concentrations are noted in different tumors making the cancer tissues more resistant to prooxidant therapies, including chemotherapy and radiotherapy. Enhancing the capacity of GSH to protect the cells from the changes in redox state or environmental toxins could be important as the cytoprotective strategy against cancer. On the contrary, depletion of GSH and related detoxification pathways could sensitize cancer cells to chemotherapy. In our investigation, content of GSH, as hydrophilic compound, was the highest in water extract. The lowest value of GSH was noted in juice. 

In the study of Zhang et al., DPPH﮲ scavenging activity of water and 95% hydroethanolic extracts from the root of *S. divaricata* significantly correlated with total phenolics content [44]. A similar situation was noted in the case of ABTS﮲^+^ scavenging activity and 80% hydromethanolic extract [45]. Our results of scavenging activities of ABTS﮲^+^ and DPPH﮲ and concentration of polyphenols are in agreement with the knowledge that the content of total phenolics is major contributor to the antioxidative activity of herbs [44,45].

The content of polyphenols in the water extract was about 5.036%. In turn, Park et al. determined in the water extract from *S. divaricata* the contents of individual phenolic acids and flavonoids, which have high antioxidant activities [46]. The content of phenolic acids was 1.369% and the content of flavonoids—1.708%. Among phenolic acids, phloroglucinol, coumarin, chlorogenic, gallic, caffeic, vanillic and *trans*-ferulic acids were quantified and among flavonoids—(−) epigallocatechin, gallocatechin and 3-hydroxyflavone. Numerous in vitro and in vivo studies showed that these polyphenols have anticancer properties. It was demonstrated that chlorogenic acid and caffeic acid decreased the viability of Caco-2 cells, arrested the cell cycle at the S phase and induced apoptosis [47]. In turn, according to Hou et al., chlorogenic acid decreased the viability of human colon cancer cells, induced the production of ROS and the cell cycle arrest at the S phase [48]. In the research on rats, chlorogenic acid showed inhibitory effect on azoxymethane-induced colon carcinogenesis [49]. Chlorogenic acid inhibited also colon cancer cell-induced lung metastasis [50]. Another phenolic acid, gallic acid, showed chemopreventive effect against 1.2-dimethylhydrazine-induced colon carcinogenesis in rats [51]. Moreover, gallic acid decreased the viability of human colon cancer cells [52]. It arrested the cell cycle at the SubG1 phase, induced the generation of ROS and apoptosis. In the study of Forester et al., gallic acid decreased the viability of human colon cancer cells, arrested the cell cycle at the Go/G1 phase and activated apoptosis [53]. Caffeic acid, in turn, decreased the viability of human colon cancer cells, increased the accumulation of the cells at the SubG1 phase of the cell cycle, ROS generation and induced apoptosis [54]. Another phenolic acid, *trans*-ferulic acid, increased intracellular ROS content in human lung cancer cells, decreased their viability, induced moderate apoptosis and moderately inhibited the cell migration [55]. The flavonoid (−) epigallocatechin decreased the viability of human colon cancer cells and other cancer cells [56,57,58] and induced apoptosis [56,58]. Another flavonoid, 3-hydroxyflavone, decreased the viability of Caco-2 cells [59].

The juice and extracts from the root of *S. divaricata* consisted mainly of carbohydrates. Their content was the highest in juice and 75% hydroethanolic extract and the smallest in the 50% hydroethanolic extract. In turn, Gao et al. demonstrated that the polysaccharides of the root of *S. divaricata* consisted of eight monosaccharides–arabinose: glucose: galactose: mannose: galacturonic acid: rhamnose: ribose: fucose, in the molar ratio of: 47.70:23.69:12.06:7.86:4.42:3.10:0.71:0.45 [60]. According to Xue-Mei, the monosaccharide constituents of *S. divaricata* were galacturonic acid: arabinose: galactose: rhamnose, in the proportion of 4.8: 2.3:1:0.15 [61]. Moreover, Wang et al. separated from the root and rhizome of *S. divaricata* two main acidic polysaccharides [62]. The first one consisted of galacturonic acid: arabinose: galactose: rhamnose, in the molar ratio of 4.8:2.3:1:0.15 and the second one of galacturonic acid: arabinose: galactose: rhamnose: xylose, in the molar ratio of 10.2:1.5:1:0.8:0.2. In turn, Zhang et al. separated *S. divaricata* polysaccharides into acid and neutral [63]. The acid polysaccharides had strong antioxidant activity, better than neutral. In vitro and in vivo studies demonstrated anticancer and immunomodulatory effects of *S. divaricata* polysaccharides. The results of Li et al. showed that these polysaccharides inhibited the growth of the implanted S180 murine sarcoma tumor and the inhibition was significantly higher when polysaccharides were used with IL-2 [64]. Moreover, K562 myelogenous leukemia cells treated with *S. divaricata* polysaccharides demonstrated decreased viability. The apoptosis was also confirmed and its percentage increased with increasing concentrations of polysaccharides [65]. Dong et al. obtained from the water extract of *S. divaricata* two polysaccharides, SDNP-1 and SDNP-2, having apparent molecular weight of 67.9 × 10^3^ Da and 5.2 × 10^3^ Da [66]. They were composed of arabinose and galactose (molar ratio of about 1:1) and deduced to be AGII-type arabinogalactans having different backbone chains. SDNP-2, but not SDNP-1, could antagonize the immunosuppression by B16F10 melanoma cells on RAW264.7 macrophages.

*In vitro* anticancer effect of ethanolic extract from *S. divaricata* root was demonstrated by Kuo et al. [67]. At the concentration of 100 μg/mL, this extract significantly suppressed the proliferation of different cancer cell lines: Raji human Burkitt’s lymphoma, HeLa human cervical adenocarcinoma, Calu-1 human lung cancer, Wish (a HeLa derivative), K562 human chronic myelogenous leukemia and Vero cells, after 72 h of treatment. Moreover, according to these authors, the polyacetylene panaxynol, isolated from *S. divaricata*, blocked the proliferation of above cells. It did not significantly change the cell viabilities measured using the test based on the membrane integrity and thus it did not exhibit direct cytotoxicity. After the incubation with panaxynol, the cells were blocked at the G0/G1 phase of the cell cycle. In addition, the authors observed that it decreased mRNA content of cyclin E, which appears to be necessary for the G1/S transition. The authors suggested that panaxynol interfered with the regulatory events required for the entry of cancer cells into the S phase of the cell cycle, which is related to the suppression of the cell proliferation. According to Chu et al., the traditional Chinese medicinal formula, containing, among others, the root of *S. divaricata*, by downregulating *STAT3* (signal transducer and activator of transcription-3) signaling pathway playing the important function in the colorectal cancer progression, effectively suppressed the tumor growth and angiogenesis [68]. The induction of apoptosis by this formula might also be a main mechanism of the inhibition of tumor growth.

The results of the study of Tai and Cheung [18] indicate the potential applicability of 70% hydroethanolic extract from the root of *S. divaricata* as an element of combination therapy. This extract showed antiproliferative activities on HL60 human acute promyelocytic leukemia, MDA-MB-468 human mammary adenocarcinoma, K562 human chronic myelogenous leukemia and MCF7 human mammary adenocarcinoma cell lines. The most susceptible were HL60 cells and the least susceptible–MCF7. The combination of the extract with chemotherapeutic drugs, camptothecin or paclitaxel, demonstrated additive antiproliferative effects on the examined cells. However, the effects depended on the cell line and the doses of the drugs and *S. divaricata* extract. The results of the authors suggest that as a result of the co-administration of *S. divaricata* extract with lower doses of chemotherapeutic agents could be achieved a similar anti-proliferative effect to that when chemotherapeutics are administered alone, at higher doses, that can demonstrate toxic side-effects.

Treatment with juice from the root of *S. divaricata*, at the concentration of 10, 1 and 0.1 mg/mL, for 24 h and 50% hydroethanolic extract from this material, at the concentrations of 1, 0.1 and 0.01 mg/mL, for 72 h resulted in a decrease of the viability of Caco-2 cells. The juice compounds of molecular weight >50 kDa turned out to reduce the viability, after 24 h of treatment. The effect of juice and 50% hydroethanolic extract against the cell viability may be assigned to the presence of the proper concentrations of chromones, furanocoumarins, polyphenols, polysaccharides and other bioactive compounds typical for this herb and their additive or synergistic properties. The juice might have contained in the fraction >50 kDa the anticancer compounds which had not been efficiently obtained in the extracts, mainly polysaccharides. LDH activity in the medium, indicating the integrity of cell membranes, did not change after 24-h incubation of the cells with juice and 50% hydroethanolic extract. Thus, they could have not exhibited direct cytotoxic activity. However, some concentrations of juice and this extract decreased LDH activity after 72 h of incubation. It may indicate the protective properties of contained chemical compounds in relation to the cell membranes. Moreover, juice and 50% hydroethanolic extract significantly increased the number of early apoptotic cells, after 24 of treatment. This extract also decreased the number of live cells, after 72 h of incubation. These prove that the bioactive compounds contained in juice and 50% hydroethanolic extract from *S. divaricata* root limited, to some extent, the viability of Caco-2 cancer cells by inducing apoptosis. There is a high probability that juice and 50% hydroethanolic extract could have also reduced cell proliferation and affect the cell cycle, that requires further research.

## 4. Materials and Methods

### 4.1. Plant Material and Preparation of Juice and Extracts

Plant raw material (roots), about 1 kg, was collected from three-year-old plants cultivated at the experimental field of the Department of Vegetable and Medicinal Plants, Warsaw University of Life Sciences (Warsaw, Poland). Seed material of *S. divaricata* used to establish experimental field plantation originated from wild growing population located in Mongolia. Underground organs were harvested in the late autumn, 2016. Voucher specimens were deposited at herbarium of the Department of Vegetable and Medicinal Plants, Warsaw University of Life Sciences.

One part (about 0.5 kg) of the fresh raw material was used to obtain juice. The roots were washed and the juice was extracted in low temperature by single-auger low-speed juicer (Hurom HZS Alpha Plus, Hurom Co., Ltd., Gimhae-si, South Korea), then it was centrifuged two times (18516× *g*, 10 min), filtered (filter paper), separated for individual assays and stored (−80 °C). 

The second part (about 0.5 kg) of the fresh roots, directly after collection of plants, was washed and dried at 50 °C, in the dark. Air-dry, powdered raw material (5 g) was extracted with 50 mL of the following solvents (a) water—to prepare water extract, (b) ethanol:water 50:50 (v/v)—to make 50% hydroethanolic extract and (c) ethanol:water 75:25 (v/v)—to prepare 75% hydroethanolic extract, using Büchi Extraction System B-811 (Büchi Labortechnik AG, Flawil, Switzerland). Soxhlet hot extraction with twenty five extraction cycles, for 5 h 10 min was used. The Soxhlet hot extracts were filtered and concentrated up to 5 mL, using a rotary evaporator (Büchi R-200 Rotavapor System, Büchi Labortechnik AG, Flawil, Switzerland). The obtained extracts were frozen at −80 °C, for 2 days and then subjected to lyophilization (Labconco FreeZone 2.5 freeze dryer, Labconco, Kansas City, MO, USA) for 2 days (−50 °C, 0.10 mbar). Dry extracts were powdered in a porcelain mortar and stored in dark vials (4 °C). 

### 4.2. Analysis of Cimifugin β-D-glucopyranoside, Cimifugin, 4′-O-Glucopyranosyl-5-O-methylvisamminol and Imperatorin by Ultra-High Performance Liquid Chromatography-Mass Spectrometry (UHPLC-MS)

The standards of above compounds (ChromaDex^®^, Irvine, CA, USA) were dissolved in methanol, in 10 mL volumetric flasks, according to ChromaDex^®^ Tech Tip 0003—Recovery & Dilution Procedures [69] and used as standard stock solutions. A mixture of those standards was prepared by mixing 200 μl of each standard stock solution with 800 μl of water.

Juice and extracts were prepared, in triplicate, by the addition to their proper amount (~5280 mg of juice (fresh matter) and ~250 mg of extracts) of proper amount of methanol (10 or 5 mL, respectively). Then, 0.5 mL of methanolic solutions were mixed with 0.5 mL of water and filtered (polyvinylidene fluoride (PVDF) syringe filters with pore size 0.22 μm, EuroClone, Pero, Italy).

An Agilent 1290 Infinity LC System (Agilent Technologies, Santa Clara, CA, USA) was used for high-resolution chromatographic separation of the analyzed compounds. The study was conducted using UHPLC coupled to ultra-high definition quadrupole time-of-flight (Q-TOF) mass spectrometry with the Agilent 1290 Infinity LC System with an Agilent 6540 Accurate-Mass Q-TOF LC/MS System (Agilent Technologies, Santa Clara, CA, USA). That system was used for identification of the peaks. The Q-TOF was operated in two modes: an initial MS-only mode, at extended dynamic range 2 Hz acquisition rate and an automatic MS/MS mode (data-dependent), with the masses of the investigated compounds. With the combined MS and MS/MS acquisition, the MS acquisition rate was 5 spectra/second and the mass range was set to 100–1000 m/z for both MS and MS/MS mode. Active exclusion mode was on and set to: exclude after 2 spectra, release after 0.05 min. The charge state of the analyzed compounds was set to 1+, 2+ and unknown. The fixed collision energies mode was chosen and set to 0, 10, 20 and 40 eV. The mass spectrometer conditions for the Dual Agilent Jet Stream electrospray ionization (ESI) source were as follows: 350 °C for the sheath gas temperature, 11 l/min for the sheath gas flow, 300 °C for the drying gas temperature, 8 l/min for the drying gas flow rate, 35 psi for the nebulizer pressure, 3500 V for the capillary voltage in positive ion mode and 1000 V for the nozzle voltage. The fragmentor voltage was set to 135 V and all other mass spectrometer parameters remained at autotune conditions. Chromatographic separations were performed on a C18 column (2.1 × 150 mm, particle size 3 μm, LumiSep, Zgierz, Poland), at 40 °C and flow rate of 0.3 mL/min. Solvents consisted of: A—50 mL methanol, 950 mL water, 1 mL formic acid, B—800 mL methanol, 200 mL water, 1 mL formic acid. All components were of HPLC grade and were purchased from VWR International (Radnor, PA, USA). Analyses were performed according to the gradient settings: 100% A for 25 min, 100% B for 2 min, 100% B for 0.01 min, 100% A for 7.99 min. Injection volume was 5 μL throughout the chromatographic experiments and UV detection was at 254 nm for quantitative analysis. Amounts of four investigated compounds were calculated by comparing peak area for standard mixture and corresponding peak area for identified compound of interest in analyzed extracts. Each of three repeats (*n* = 3) of examined samples was injected twice and peaks from the second injection were integrated. The results were expressed in μg of the examined compound per g of juice (fresh matter) or extract. 

### 4.3. Determination of Indicators of Redox State

#### 4.3.1. Ferric-Reducing Antioxidant Power

Ferric-reducing antioxidant power of juice and water, 50% and 75% hydroethanolic extracts from *S. divaricata* roots was assayed by the modified method of Oyaizu [70,71]. The method consists in reduction of Fe^3+^, present in stoichiometric excess in relation to antioxidants, due to donation of electrons by these compounds. The increase in absorbance is observed as the reduction capability increases. Juice and extracts were homogenized in deionized water and centrifuged (1600× *g*, 10 min). 2.5 mL of supernatants were mixed with 2.5 mL of 0.2 M sodium phosphate buffer (pH 6.6) and 2.5 mL of 1% potassium ferricyanide. The probes were incubated in a water bath (50 °C, 20 min) and 2.5 mL of 10% trichloroacetic acid (TCA) was added. The samples were centrifuged (3000× *g*, 5 min) and 0.4 mL of supernatants were mixed with 0.4 mL of deionized water and 160 μl of 0.1% ferric chloride. The absorbance was recorded at 700 nm, using microplate reader (Infinite M200, Tecan, Männedorf, Switzerland). The standard curve was constructed by applying different concentrations (0-100 μM) of TROLOX ((±)-6-hydroxy-2,5,7,8-tetramethylchromane-2-carboxylic acid, a water-soluble vitamin E analog). The analysis was carried out in six repeats (*n* = 6). 

#### 4.3.2. 2.2′-Azino-bis(3-ethylbenzthiazoline-6-sulfonic Acid) Radical Cation (ABTS﮲^+^) Scavenging Activity

ABTS﮲^+^, a free radical which is relatively stable, decolorizes after reduction. To determine the scavenging activity of ABTS﮲^+^ of juice and extracts from *S. divaricata* roots, they were prepared as in Section 4.3.1. and the procedure of Sun et al. with some modifications was applied [71,72]. The method consists in adding antioxidants to the ABTS﮲^+^ solution and other ABTS﮲^+^ is assessed spectrophotometrically. To prepare the ABTS reagent, 5 mL of 7 mM ABTS was combined with 88 μl of 140 mM K_2_S_2_O_8_. In order to generate radicals, the reagent was placed in a dark site (16 h, room temperature (RT)). The absorbance was adjusted to 0.70 ± 0.02 (734 nm, microplate reader Infinite M200, Tecan, Männedorf, Switzerland) by dilution of the reagent with 99.8% ethanol. The scavenging activity of the radical cation was assayed by mixing of 0.9 mL of the ABTS reagent with 0.1 mL of supernatants derived from juice and extracts from *S. divaricata* roots. After the incubation (6 min, RT), the absorbance was read. The standard curve was constructed using TROLOX (as in Section 4.3.1.), *n* = 5.

#### 4.3.3. 2.2-Diphenyl-1-picrylhydrazyl Radical (DPPH﮲ ) Scavenging Activity

To determine the scavenging activity of DPPH﮲ of juice and extracts derived from the roots of *S. divaricata* (prepared as in Section 4.3.1.), the modified procedure of Li et al. was used [71,73]. In this method, the antioxidants provide a hydrogen or electron to the DPPH﮲ unpaired electron and a decrease in absorbance is observed, proportional to rise in the DPPH non-radical form. 0.2 mM DPPH﮲ solution in absolute methanol was combined with the supernatants derived from juice and extracts from *S. divaricata* roots (2:1, v/v). After the incubation (30 min, without light access), the samples were centrifuged (15,000× *g*, 10 min), then the supernatant absorbance was measured at 517 nm (microplate reader Infinite M200, Tecan, Männedorf, Switzerland). The standard curve was constructed as in Section 4.3.1, *n* = 6.

#### 4.3.4. Glutathione

Glutathione (GSH), an ubiquitous thiol tripeptide, constitutes almost 97% of non-protein thiol compounds in cells. By determination of non-protein -SH groups in deproteinized by TCA samples, GSH is determined quantitatively. The spectrophotometric method for the determination of non-protein -SH groups is based on the Ellman’s method, in which 5,5′-dithiobis(2-nitrobenzoic acid) (DTNB, Ellman’s reagent) is reduced by thiol compounds with the formation of colored 2-nitro-5-mercaptobenzoic acid, with a maximum of absorbance at 412 nm [74]. Juice and extracts from *S. divaricata* roots were homogenized in 0.1 M phosphate buffer pH 7.4 and centrifuged (1600× *g*, 10 min). To deproteinize, to 1.5 mL of supernatants was added 78.96 μl of 50% TCA and samples were centrifuged (3000 rpm, 5 min). Then, directly on a 96-well plate, 25 μl of deproteinized supernatants were mixed with 200 μl of 0.2 M phosphate buffer pH 8.0 and with 25 μl of 6 × 10^−3^ M DTNB. The absorbance was recorded using microplate reader (Infinite M200, Tecan, Männedorf, Switzerland). The standard curve was constructed by applying different concentrations (0–75 nmol/mL) of GSH standard in 2.5% TCA, *n* = 6. 

### 4.4. Determination of Total Carbohydrates, Protein and Fat

Content of total carbohydrates in juice and extracts from *S. divaricata* roots was measured by the method using phenol and sulfuric acid [75]. Glucose was used to construct a standard curve and the absorbance was recorded at 490 nm, *n* = 3.

Concentration of total protein in juice and extracts was determined using the Bradford method and the standard was bovine serum albumin (BSA) [76], *n* = 6. 

Content of total lipids in juice and 50% hydroethanolic extract was measured using the Folch method [77], *n* = 2. 

### 4.5. Determination of Polyphenols

The method for determining the total amount of polyphenols in a sample, the Folin-Ciocalteu method, involves the measurement of the absorbance of the complex resulting from the reduction of salts of hetero polyacids, phosphomolybdic and phosphotungstic, so-called Folin-Ciocalteu reagent [78]. During the reaction Mo (VI) ions are reduced to Mo (V), which results in a blue color originating from [PMoW_11_O_40_]^4−^. To determine the concentration of polyphenols, juice and extracts derived from the roots of *S. divaricata* were prepared as in Section 4.3.1. Then, 0.5 mL of supernatants were mixed with 2.5 mL of Folin-Ciocalteu reagent (1:10 diluted with deionized water). After 2 min of incubation, the samples were combined with 2 mL of 7.5% Na_2_CO_3_. After incubation in a water bath (50 °C, 10 min), the absorbance was measured at 760 nm, in a cuvette spectrophotometer (Spectronic 20D, Milton Roy, Rochester, NY, USA). The standard curve was constructed by applying different concentrations (0–100 μg/mL) of quercetin, *n* = 3. 

### 4.6. Preparation of Juice and Extracts and Their Fractions for Cell Culture Tests

Before each cell culture test, water, 50% and 75% hydroethanolic extracts from *S. divaricata* roots were dissolved in deionized water, at the concentration of 10 mg/mL (experiment described in Section 4.8.) or 15.9 mg/mL (other tests). The juice was diluted to obtain the same concentrations as the extracts, analogically (concentrations of juice concern its dry matter (dry matter of undiluted juice = 159 mg/mL)). Then, juice and extracts were subjected to centrifugation (1600× *g*, 10 min) and the supernatants were collected. 

Considering the results of the experiment described in Section 4.8., part of juice and 50% hydroethanolic extract (159 mg/mL) was filtered using PVDF syringe filters (0.22 μm, EuroClone, Pero, Italy). Then, juice and 50% extract were fractionated in terms of molecular weight, by applying ultra centrifugal filter devices containing the membrane made of regenerated cellulose (Merck Millipore, Burlington, MA, USA), that included 3, 10 or 50 kDa cutoffs, in accordance with the manufacturer’s recommendations regarding centrifugation time and g-force. Four fractions were acquired: >50 kDa (>50 K), 10-50 kDa (10–50 K), 3–10 kDa (3–10 K) and <3 kDa (<3 K).

Before cell culture tests, juice, extracts and fractions were sterilized (PVDF syringe filters, 0.22 μm, EuroClone, Pero, Italy), under the biological safety cabinet (TopSafe^TM^ 1.2, class II, BIOAIR, Pavia, Italy). Their appropriate decimal dilutions were prepared for individual tests, using sterile deionized water. 

### 4.7. Caco-2 Cell Culture

Human epithelial colorectal adenocarcinoma (Caco-2) cell line (ECCC, 55 passage, Sigma-Aldrich, St. Louis, MO, USA) was cultured in polystyrene plates intended for adherent cell culture (for viability and membrane integrity tests—in 96-well plates, at a density of 1 × 10^4^ cells/100 μl and for types of cell death tests—in 6-well plates, at a density of 0.75 × 10^5^ cells/1.5 mL) in Minimum Essential Medium (MEM) with 2 mM L-glutamine (Thermo Fisher Scientific, Waltham, MA, USA), 10% fetal bovine serum (FBS, Thermo Fisher Scientific, Waltham, MA, USA), 1% non-essential amino acids (NEAA, Thermo Fisher Scientific, Waltham, MA, USA) and 1% antibiotic-antimycotic (Thermo Fisher Scientific, Waltham, MA, USA) [71]. The cells were placed in a CO_2_ incubator (INCO 108 med, Memmert GmbH + Co. KG, Schwabach, Germany) at 37 °C (5% CO_2_, 95% relative humidity). After incubation for 24 h and reaching about 70% confluence, they were starved overnight in MEM (Thermo Fisher Scientific, Waltham, MA, USA) with 1% FBS (Thermo Fisher Scientific, Waltham, MA, USA) and 1% antibiotic-antimycotic (Thermo Fisher Scientific, Waltham, MA, USA) [71]. 

### 4.8. Effect of Juice and Extracts on Cell Viability

Ninety μL of fresh medium (MEM with 1% FBS and 1% antibiotic-antimycotic, see Section 4.7.) and 10 μL of juice, water, 50% hydroethanolic extract and 75% hydroethanolic extract from *S. divaricata* roots, at the concentrations of 10, 1, 0.1 and 0.01 mg/mL were added to the cells. The same volumes of sterile deionized water were introduced into the control cells. Various additional controls were included. After incubation (CO_2_ incubator INCO 108 med, Memmert GmbH + Co. KG, Schwabach, Germany; 37 °C, 5% CO_2_, 95% relative humidity) for 24 h and 72 h, the MTT (methylthia-zolyldiphenyl-tetrazolium bromide) test was carried out by the modified procedure of Tada et al. [79]. A yellow solution of MTT is converted by mitochondrial dehydrogenases of live cells to dark blue, water-insoluble MTT formazan. In brief, 15 μl of MTT reagent (Sigma-Aldrich, St. Louis, MO, USA) in PBS (phosphate-buffered saline) at 5 mg/mL was introduced into the cells and the plates were incubated (37 °C, 4 h). Then, 100 μl of lysis buffer (10% sodium dodecyl sulfate in 0.01 M HCl) was introduced, plates were incubated (37 °C, overnight) and the absorbance was recorded at 570 nm using microplate reader (Infinite M200, Tecan, Männedorf, Switzerland), *n* = 5. 

### 4.9. Effect of Fractions of Juice and 50% Hydroethanolic Extract on Cell Viability

Ninety μL of fresh medium and 10 μL of four fractions: >50 kDa (>50 K), 10–50 kDa (10–50 K), 3–10 kDa (3–10 K) and <3 kDa (<3 K) of juice from *S. divaricata* roots, at the concentrations of 15.9, 15.9 × 10^−1^, 15.9 × 10^−2^ and 15.9 × 10^−3^ mg/mL or the same concentrations of the same fractions of 50% hydroethanolic extract (the concentration of the extract before fractionation corresponded to the dry matter of the juice) were added to the cells. The same controls as in Section 4.8. were included. After 24 h of incubation of the cells with juice and extract fractions, the MTT test was performed, as in Section 4.8. *n* = 5. 

### 4.10. Effect of Juice and 50% Hydroethanolic Extract on Membrane Integrity

Damage to the cell membrane releases a cytosolic enzyme, lactate dehydrogenase (LDH) into the medium. It can be quantified using a coupled enzymatic reaction. LDH is a catalyst of the conversion of lactate to pyruvate via NAD^+^ reduction to NADH. Then, diaphorase uses NADH to reduce INT (terazolium salt) to a red formazan which content is recorded at 490 nm. The LDH test was performed following the recommendations of the manufacturer of the commercial kit used (Thermo Fisher Scientific, Waltham, MA, USA). To the cells were added 100 μL of fresh medium and 10 μL of juice or 50% hydroethanolic extract from *S. divaricata* roots, at the concentrations of 15.9, 15.9 × 10^−1^, 15.9 × 10^−2^, 15.9 × 10^−3^, 15.9 × 10^−4^ and 15.9 × 10^−5^ mg/mL. The same volumes of sterile deionized water were introduced into the control cells. Different additional controls were included. After incubation (CO_2_ incubator INCO 108 med, Memmert GmbH + Co. KG, Schwabach, Germany; 37 °C, 5% CO_2_, 95% relative humidity) for 24 h and 72 h, LDH activity in cell culture medium was determined and expressed as % of maximum LDH activity (in medium after lysis of the cells), *n* = 6. 

### 4.11. Effect of Juice and 50% Hydroethanolic Extract on Types of Cell Death

To the cells were added 1.5 mL of fresh medium and 150 μL of juice or 50% hydroethanolic extract from *S. divaricata* roots, at the concentrations of 15.9 × 10^−1^ and 15.9 × 10^−2^ mg/mL. The same volumes of sterile deionized water were introduced into the control cells. After incubation of the cells (CO_2_ incubator INCO 108 med, Memmert GmbH + Co. KG, Schwabach, Germany; 37 °C, 5% CO_2_, 95% relative humidity) for 24 h (15.9 × 10^−1^ mg/mL) and for 72 h (15.9 × 10^−2^ mg/mL), the types of cell death were evaluated according to the instructions of the manufacturer of the commercial kit for flow cytometry with Alexa Fluor^®^ 488 Annexin V and propidium iodide (PI) (Thermo Fisher Scientific, Waltham, MA, USA). PI is a fluorescent dye which stains dead cells binding to the nucleic acids and Annexin V conjugated to fluorophore Alexa Fluor^®^ 488 binds to phosphatidyl serine exposed on the outer surface of the cell membrane of the apoptotic cells. Live cells are unstained, Annexin V-positive cells are considered as early apoptotic, PI/Annexin V-positive cells as late apoptotic and PI-positive cells as necrotic. The cells were harvested by trypsinization and washed twice in cold PBS. After centrifugation and removal of the supernatant, the cell pellets were resuspended in 100 μl of Annexin-binding buffer. Then, 5 μl of Alexa Fluor^®^ 488 Annexin V and 1 μl of PI working solution were added to each cell suspension. The cells were incubated for 15 min (RT). Then, 400 μl of Annexin-binding buffer was added, the samples were gently mixed and kept on ice until they were introduced into the flow cytometer. The stained cells were analyzed by BDFACSCalibur™ flow cytometer (Becton Dickinson, Franklin Lakes, NJ, USA), intensity of fluorescence emission was measured using FL1 channel for Alexa Fluor^®^ 488 at 530 nm and FL2 for PI at 575 nm, using excitation at 488 nm. Ten thousand (24-h test) or forty thousand events (72-h test) were recorded per sample. Plots were generated using Flowing Software 2.5.1 (Perttu Terho, Turku, Finland), *n* = 6. 

The cells were imaged by an inverted light microscope DMi8 equipped with a MC190 HD camera, using the LAS V4.10 software (Leica, Wetzlar, Germany).

### 4.12. Statistical Analysis

The results are expressed as the mean ± the standard error of the mean (SEM). The results of the part concerning the chemical composition were subjected to a one-way analysis of variance (ANOVA) and the means were compared by the Tukey’s post-hoc test. Statgraphics Centurion software (StatPoint Technologies, Inc., Warrenton, VA, USA) was used. The results of tests on cells were subjected to ANOVA, the mean values for juice and extract treated groups were compared to deionized water treated group by the Dunnett’s post-hoc test. Prism 5 software (GraphPad Software Inc., San Diego, CA, USA) was applied. The difference at *p* < 0.05 between the means was considered statistically significant.

## 5. Conclusions

The examined chromones (cimifugin β-D-glucopyranoside, cimifugin and 4′-O-gluco- pyranosyl-5-O-methylvisamminol) and furanocoumarin imperatorin were contained in juice from *S. divaricata* root in the largest concentrations. Juice and extracts, due to the presence of antioxidants, that were characterized by the electron or hydrogen donor properties to neutralize free radicals, have the ability to reduce the oxidative stress in the organism and may be a valuable chemopreventive diet components decreasing the colon cancer risk. The content of GSH, responsible for maintenance of the intracellular redox balance, as hydrophilic compound, was the highest in water extract. The major contributor to the antioxidative activity of juice and extracts were the most probably polyphenols. The concentration of carbohydrates was the highest in juice and 75% hydroethanolic extract. The decrease of the viability of the colon cancer cells Caco-2, after the application of juice and 50% hydroethanolic extract from the root of *S. divaricata* was affirmed in the present work for the first time. This effect could be related to presence of chromones, furanocoumarins, polyphenols, polysaccharides and other bioactive compounds typical for *S. divaricata*, at proper concentrations, and their additive or synergistic properties. The observed reduction of the cell viability after the use of the fraction of juice with the molecules >50 kDa may be assigned to the presence of anticancer compounds which were not efficiently obtained in the extracts, mainly polysaccharides. Some chemical compounds of juice and 50% hydroethanolic extract could have exhibited the protective properties in relation to the cell membranes. The viability of Caco-2 cancer cells after the application of juice and 50% hydroethanolic extract was decreased to some extent by inducing apoptosis. The knowledge of further exact mechanisms of anticancer activity of the examined natural products from *S. divaricata* will allow to consider the use of juice and 50% hydroethanolic extract as part of combination therapy of colon cancer. The conventional treatment in combination with these natural products may affect more signaling pathways decreasing the development of resistance to chemotherapeutics, increase the sensitivity to anticancer drugs and minimize adverse effects of therapy.

## Figures and Tables

**Table 1:** Concentration of total carbohydrates, protein and fat and polyphenols in juice (J), water extract (W E), 50% hydroethanolic extract (50% E) and 75% hydroethanolic extract (75% E) from *Saposhnikovia divaricata* root ^1^.

Compound	J	W E	50% E	75% E	*p*
Total carbohydrates (% DM)	71.76 ± 1.72 ^BC^	64.64 ± 3.04 ^B^	47.87 ± 0.72 ^A^	77.92 ± 1.52 ^C^	< 0.0001
Total protein (% DM)	5.13 ± 0.40 ^B^	0.21 ± 0.02 ^A^	0.75 ± 0.00 ^A^	< 0.1	< 0.0001
Total fat (% DM)	5.44 ± 0.11 ^A^	ND	14.90 ± 0.00 ^B^	ND	0.0001
Polyphenols (μg quercetin/mg DM)	25.31 ± 0.14 ^A^	50.36 ± 0.16 ^B^	84.14 ± 0.16 ^D^	66.68 ± 0.28 ^C^	< 0.0001

^1^ Data are expressed as mean ± standard error of the mean. Statistically significant effect: values of one parameter without common superscript (A,B,C,D) are significantly different (*p* < 0.01). *n* = 3 (total carbohydrates and polyphenols), *n* = 6 (total protein), *n* = 2 (total fat). ND—not determined, DM—dry matter.
